# Genetic basis of the very short life cycle of ‘Apogee’ wheat

**DOI:** 10.1186/s12864-017-4239-8

**Published:** 2017-10-31

**Authors:** Genqiao Li, Rungravee Boontung, Carol Powers, Vikas Belamkar, Tianrong Huang, Fang Miao, P. Stephen Baenziger, Liuling Yan

**Affiliations:** 1 Department of Plant and Soil Sciences, Oklahoma State University, Stillwater, OK74078 USA; 2 Department of Agronomy and Horticulture, University of Nebraska-Lincoln, Lincoln, NE 68583 USA; 3 Wheat, Peanut and Other Field Crops Research, USDA-ARS, 1301 N Western RD, Stillwater, OK 74075-2714 USA; 4 Institute of Grain Crops, Xinjiang Academy of Agricultural Sciences, Urumqi, 830091 People’s Republic of China; 5 College of Life Science, Northwest A & F University, Yangling, Shaanxi 712100 People’s Republic of China

**Keywords:** Apogee wheat, Flowering genes, Vernalization, Photoperiod, *VRN1*

## Abstract

**Background:**

‘Apogee’ has a very short life cycle among wheat cultivars (flowering 25 days after planting under a long day and without vernalization), and it is a unique genetic material that can be used to accelerate cycling breeding lines. However, little is known about the genetic basis of the super-short life of Apogee wheat.

**Results:**

In this study, Apogee was crossed with a strong winter wheat cultivar ‘Overland’, and 858 F_2_ plants were generated and tested in a greenhouse under constant warm temperature and long days. Apogee wheat was found to have the early alleles for four flowering time genes, which were ranked in the order of *vrn-A1* > *VRN-B1* > *vrn-D3* > *PPD-D1* according to their effect intensity. All these Apogee alleles for early flowering showed complete or partial dominance effects in the F_2_ population. Surprisingly, Apogee was found to have the same alleles at *vrn-A1a* and *vrn-D3a* for early flowering as observed in winter wheat cultivar ‘Jagger.’ It was also found that the *vrn-A1a* gene was epistatic to *VRN-B1* and *vrn-D3*. The dominant *vrn-D3a* alone was not sufficient to cause the transition from vegetative to reproductive development in winter plants without vernalization but was able to accelerate flowering in those plants that carry the *vrn-A1a* or *Vrn-B1* alleles. The genetic effects of the vernalization and photoperiod genes were validated in Apogee x Overland F_3_ populations.

**Conclusion:**

*VRN-A1, VRN-B1*, *VRN-D3*, and *PPD-D1* are the major genes that enabled Apogee to produce the very short life cycle. This study greatly advanced the molecular understanding of the multiple flowering genes under different genetic backgrounds and provided useful molecular tools that can be used to accelerate winter wheat breeding schemes.

**Electronic supplementary material:**

The online version of this article (10.1186/s12864-017-4239-8) contains supplementary material, which is available to authorized users.

## Background

Two wheat (*Triticum aestivum* L. 2n = 6× = 42, AABBDD) cultivars, ‘USU-Apogee’ and ‘USU-Perigee’, were developed and released in 1996 by the Utah Agricultural Experiment Station at Utah State University (USU) in cooperation with the National Aeronautics and Space Administration (NASA) for improved food crops for the Advanced Life Support System (ALSS) in a space station [1]. Apogee is named for the point in an orbit farthest from the earth, while Perigee is named for the point in an orbit closest to the earth. The two wheat cultivars were used to regenerate oxygen, purify water, and produce food [2]. Due to restricted space, one of the major requirements for a wheat cultivar adapted to grow in the ALSS food system is a short cycle life.

Apogee can flower 25 days after planting when grown under constant conditions (23 °C warm temperature and continuous light) [3], which is the shortest life cycle in wheat in the world. Under the same conditions, Perigee flowers 30 days after planting and also displays an extremely rapid development rate. In addition to the unique rapid life cycle, the two hard red spring wheat cultivars have several other properties suitable for the space station, including dwarf stature that allow the plants to grow on shelves, resistance to the calcium-induced leaf tip necrosis that occurs in rapid growth conditions, and high grain yield, particularly in high temperature environments [2, 4, 5]. Therefore, Apogee and Perigee are elite genetic materials for breeding rapid cycling lines.

The exceptionally short life of Apogee and Perigee is mainly due to their spring growth habit. Unlike winter wheat varieties that require an extended exposure to low temperatures to accelerate flowering (vernalization, VRN), Apogee and Perigee flower under temperature-photoperiod controlled conditions and without vernalization. The developmental process of wheat is mainly controlled by genes in three pathways, including vernalization response, photoperiod response (PPD), and earliness per se (EPS) [5–7]. Among thestrongest effective mechanism underlying wheat flowering is vernalization that distinguishes spring wheat and winter wheat [8, 9]. Major genes responsible for the difference in vernalization requirement between the two distinct types have been cloned, including *VRN-1* [10], *VRN-3* [11], and *VRN-D4* [12] whose expression is promoted by low temperature, and *VRN-2* [13] that is repressed by low temperature. The wheat *PPD* genes for photoperiod sensitivity were isolated [14] based on77 the sequence of the orthologous *PPD-H1* gene in barley [15]. No *EPS* gene for earliness per se has been cloned from either spring or winter wheat cultivars [7, 16].

Apogee and Perigee are cultivars of common hexaploid wheat that has three homoeologous genomes A, B, and D, forming a series of homoeologous genes, such as *VRN-A1*, *VRN-B1*, and *VRN-D1* on chromosomes 5AL, 5BL, and 5DL [17]. The homoeologous genes have high identity in sequence in genetic backgrounds in different wheat cultivars, making it complicated to genotype a specific cultivar for three homoeologous genes. The existence of multiple alleles in a wheat homoeologous gene could even make it more complicated to genotype a specific cultivar. *VRN1* was first cloned from diploid wheat *T. monococcum* (2*n* = 2*×* = 14, genome A^m^A^m^) [10], and five spring alleles of *Vrn-A* ^*m*^ *1*, varying in deletion lengths (alleles *Vrn-A* ^*m*^ *1a*, *Vrn-A* ^*m*^ *1b*, *Vrn-A* ^*m*^ *1g*, *Vrn-A* ^*m*^ *1f*), are involved in a so-called CArG-box recognition site [18–20], compared with the recessive *vrn-A* ^*m*^ *1* allele for winter wheat. A recent study reports a novel allele, *Vrn-A1u*,that has a small deletion in the promoter region in diploid *T. urartu* (2*n* = 2*×* = 14, genome AA) [21]. Two alleles that have different deletions in the promoter (*Vrn-A1d* and *Vrn-A1e*) and one allele that has a large deletion in intron one (*Vrn-A1c*) were observed in tetraploid wheat [18]. In hexaploid spring wheat, some cultivars have a deletion in the promoter (*Vrn-A1b*), but more than half of varieties have the *Vrn-A1a* allele that has a miniature inverted-repeat transposable element (MITE) inserted in its promoter (*Vrn-A1a*) (MITE_VRN) [18]. The MITE_VRN can be recognized by microRNA *Ta*miRNA1123 [22]. While the regulatory elements are found in the promoter or intron one in spring wheat, single nucleotide polymorphisms (SNPs) found in coding regions explain quantitative variation in vernalization requirements in winter wheat. While the *vrn-A1a* allele in weak winter wheat ‘Jagger’ requires less cumulative low temperatures and the *vrn-A1b* allele in the stronger winter wheat ‘2174’ requires more cumulative low temperatures to reach a vernalization saturation point, the two *vrn-A1* alleles rely on variation not in the promoter or intron but at the protein level [23].

Compared with the many *VRN-A1* alleles as reviewed above, *VRN-B1* and *VRN-D1* have fewer observed haplotypes. *Vrn-B1* in spring wheat has a deletion in intron one in hexaploid wheat and tetraploid *T. turgidum* ssp. *durum* [10, 18, 24] or a 5.6 kb retrotransposable element (Retrotrans_VRN) in the 5′-untranslated region (UTR) in tetraploid *T. turgidum* subsp. *carthlicum* [25]. *Vrn-D1* in spring wheat has a large deletion in intron one in hexaploid wheat [24].

Relative to the multiple alleles in the *VRN1* genes, other genes known to regulate flowering time have fewer haplotypes. In spring wheat cultivars, the *Vrn-B3* allele has the insertion of a retroelement in its promoter region in the cultivar Hope [11], and *Vrn-A3* and *Vrn-D3* have variation in the non-coding intronic region [26]. In winter wheat cultivars, allelic variation between *vrn-D3*a for early flowering and *vrn-D3b* for late flowering is due to a (G)_3or4_ polymorphism in exon 3 that results in a frame shift involving 81 amino acids [26–28]. In both spring wheat and winter wheat, allelic variation between *PPD-D1b* for insensitivity to photoperiod causing early flowering and *vrn-D3a* for sensitivity to photoperiod causing late flowering is due to an indel polymorphism in the promoter of *PPD-D1* [14, 28]. Orthologous genes in hexaploid wheat for *VRN-A2*, *VRN-B2*, and *VRN-D2* have been isolated in common wheat, but no association has been observed between a deletion in *VRN-B2* and flowering time in common wheat [29, 30].

The aim of this research was to test if the super-short life of Apogee wheat is associated with genes/alleles known to regulate flowering time in common wheat. The association of the gene alleles with segregation of flowering time was tested in a large F_2_ population generated by using a winter wheat cultivar ‘Overland’ to cross with Apogee, and the observed effects of the genes/alleles were validated in F_3_ populations.

## Results

### Segregation of flowering time in F_2_ population

When tested under long-day conditions and without vernalization across the experiment, Apogee flowered 29 days after planting, whereas Overland did not flower for 3 months without vernalization. When treated with low temperature at 4 °C and under long-day conditions for six weeks, Overland had earlier flowering 34 days, confirming that Overland is a winter wheat cultivar. F_1_ plants that were generated from a cross between Apogee and Overland flowered 45 days after planting, indicating that those genes for early flowering time in Apogee were primarily dominant to those for late flowering time in Overland. A population of 858 F_2_ plants generated from the selfing of the F_1_ plants were tested, and these F_2_ plants on average flowered 48.2 days after planting, within a range from 25 days to 264 days.

**Fig. 1:**
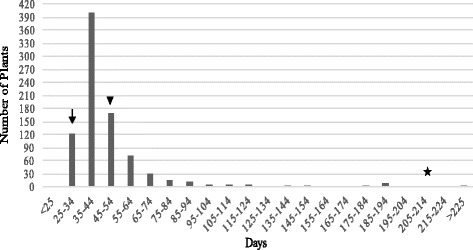
Frequency distributions of plants for flowering time in F_2_ population of Apogee X Overland. Black arrow indicates Apogee, star indicates Overland, and black triangle indicates F_1_ plant of Apogee X Overland

The flowering time of the Apogee x Overland F_2_ plants was continuous, and it had no clear cut-off for flowering time to distinguish between ‘spring’ wheat and ‘winter’ wheat. Plant number distribution of the 858 F_2_ plants showed a bias to early flowering time (Fig. 1), indicating that several genes for early flowering time are present in the population. Molecular markers for four flowering genes were identified for the causal basis of the rapid cycling of Apogee.

### Allelic variation in *vrn-A1* and its association with flowering time

Apogee and Overland showed no difference in the *VRN-A1* promoter region by using primers VRN1AF/VRN1R, and both carry the winter *vrn-A1* allele. Apogee and Overland showed no difference in intron one, Ex1/C/F//Intr1/A/R3 for the non-deletion produced no product but Intr1/C/F//Intr1/AB/R amplified products of 1068 bp, the expected size for the non-deletion region. The sequencing results indicated that both Apogee and Overland carried a winter *vrn-A1* allele in the promoter region and intron one.

**Fig. 2:**
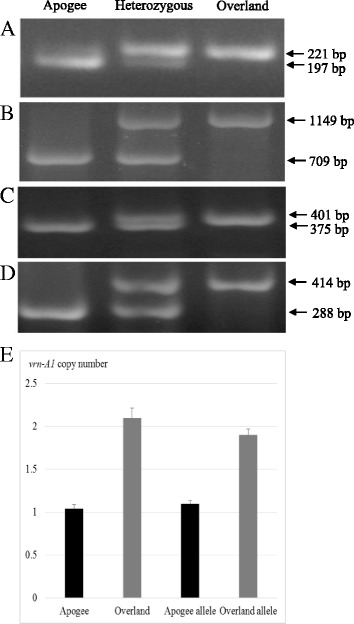
Molecular markers for *vrn-A1*, *VRN-B1*, *vrn-D3*, and *PPD-D1*. **a** PCR marker for *vrn-A1*. PCR products were amplified using primers VRN-A1F7B/VRN-A1R7, digested with restriction enzyme *Sph* I, and electrophoresed in a 2% agarose gel. Due to a SNP in exon 7, the *vrn-A1a* allele showed a band of 197 bp (and a band of 24 bp out of the gel), and the *vrn-A1b* allele showed a band of 221 bp. **b** PCR marker for *VRN-B1*. PCR reactions were performed using the forward primer Intr1/B/F to pair with two reverse primers Intr1/B/R3 and Intr1/B/R4, and PCR products were electrophoresed in a 1% agarose gel. **c** PCR marker for *vrn-D3.* PCR products were amplified using primers VRN-D3F6 and VRN-D3R8, digested with restriction enzyme *Nco* I, and electrophoresed in a 2% agarose gel. **d** PCR marker for *PPD-D1*. PCR reactions were performed using the forward primer PPD-D1_F to pair with two reverse primers PPD-D1_R1 and PPD-D1_R2, and PCR products were electrophoresed in a 1% agarose gel. **e** RT-PCR for vrn-A1 copy number. Genomic DNA samples for Apogee and two plants having the Apogee allele (A) and Overland and two plants the Overland allele (B) were tested. Copy number is shown using the values calculated by the 2^(−ΔΔCT)^ method, where CT is the threshold cycle. Bar indicates standard error

However, Apogee and Overland were detected to have differences in coding region by using primers VRN-A1F4/VRN-A1R42 for a SNP in exon 4 and VRN-A1F7B/VRN-A1R7 for a SNP in exon 7 to amplify the *vrn-A1* gene and the restriction enzyme *Sph* I to digest the PCR products(Fig. 2a). The sequencing results indicated that Apogee was found to carry the same *vrn-A1a* allele for early flowering as observed in Jagger; whereas Overland was found to have the same *vrn-A1b* allele for late flowering as observed in 2174. Real time PCR result showed that Overland had two copies of *vrn-A1* but Apogee had only one copy (Fig. 2e). This result confirmed that Apogee had the same allele as Jagger carrying one copy *vrn-A1a* and Overland had the same allele as 2174 carrying two copies *vrn-A1b*. The marker for SNP in exon 7 of *vrn-A1* was used to genotype the 858 individual plants of Apogee × Overland F_2_ population.

The plants that carried the homozygous Apogee *vrn-A1a* allele flowered, on the average, 36.8 days after planting, whereas the plants that carried the homozygous Overland *vrn-A1b* allele flowered 82.5 days after planting (Table 1). The difference between the plants carrying the homozygous Apogee *vrn-A1a* allele and the plants carrying the heterozygous *vrn-A1a/vrn-A1b* alleles was 6.5 days for flowering time (36.8 days vs. 43.3 days), but the difference between the plants carrying the homozygous Overland *vrn-A1b* allele and the plants carrying the heterozygous *vrn-A1a/vrn-A1b* alleles was 39.2 days for flowering time. These results indicated that the Apogee *vrn-A1a* allele for early flowering was partially dominant to the Overland *vrn-A1b* allele for late flowering.

**Table 1:** Comparative effects of allelic variation at *vrn-A1*, *VRN-B1*, *vrn-D3*, and *PPD-D1* on heading date

Gene	Allele^a^	No. plants	Heading date (days)	T-test^b^
*vrn-A1*	Apogee	192	36.8	a
	H	528	43.3	b
	Overland	138	82.5	c
*VRN-B1*	Apogee	228	42.5	a
	H	409	46.1	b
	Overland	221	57.9	c
*vrn-D3*	Apogee	221	44.5	a
	H	425	47.5	a
	Overland	212	53.4	b
*PPD-D1*	Apogee	247	45.5	a
	H	400	48	a
	Overland	211	51.8	b

^a^ *Apogee* homozygous Apogee allele, *Overland* homozygous Overland allele, *H* heterozygous alleles

^b^Values followed by the same letter are not significantly different at *P* < 0.05, and values followed by a different letter are significantly different at *P* < 0.05

### Allelic variation in *VRN-B1* and its association with flowering time

Apogee and Overland showed allelic variation in intron one of *VRN-B1*. As shown in Fig. 2b, PCRs with primers Intr1/B/F//Intr1/B/R3 amplified products with 709 bp from Apogee, but Intr1/B/F//Intr1/B/R4 amplified products of 1149 bp from Overland, indicating that the deletion was present in Apogee but not in Overland.

The average flowering time in plants homozygous for the Apogee deletion allele was 42.5 days, which was 15.4 days earlier than plants homozygous for the Overland allele (57.9 days) (Table 1). The plants carrying the homozygous Apogee deletion allele had a significantly earlier flowering time (3.6 days, *p* = 7.03E-08) than the plants heterozygous for *VRN-B1*. The plants heterozygous for *VRN-B1* also were significantly earlier (11.8 days, *p* = 3.72E-07) than plants homozygous for the Overland allele. These results indicated that the Apogee deletion allele for early flowering (referred to *Vrn-B1* hereafter) was partially dominant to the Overland allele for later flowering (referred to *vrn-B1* hereafter).

### Allelic variation in *vrn-D3* and its association with flowering time

Apogee and Overland showed allelic variation in exon 3 of *vrn-D3* (Fig. 2c)*.* By using primers VRN-D3-F6/VRN-D3-R8 to amplify the *VRN-D3* gene and following digestion with the restriction enzyme *Nco*I, Apogee produced two fragments, visible 375 bp and invisible 27 bp (out of the gel). The Apogee allele at *VRN-D3* was the same as the Jagger *vrn-D3a* allele for early flowering time and physiological maturity; whereas Overland produced a single fragment with 401 bp due to a deletion of 1 bp, which was the same as the 2174 *vrn-D3b* allele for late flowering time and physiological maturity.

Plants homozygous for the Apogee *vrn-D3a* allele had an average flowering time of 44.5 days, which was 8.9 days earlier than plants homozygous for the Overland *vrn-D3b* allele (53.4 days). The difference between the homozygous Apogee *vrn-D3a* allele and heterozygous *vrn-D3a/vrn-D3b* alleles (47.5 days) was not significant (*p* = 0.107), but the difference between the heterozygous *vrn-D3a/vrn-D3b* alleles and homozygous Overland *vrn-D3b* allele was significant (*p* < 0.001). These results indicated that the Apogee *vrn-D3a* allele for early flowering was dominant to the Overland *vrn-D3b* allele for late flowering.

### Allelic variation in *PPD-D1* and its association with flowering time

The diagnostic marker used to identify allelic variation in the promoter indel region of the *PPD-D1* gene showed that Apogee carried the photoperiod insensitive allele *PPD-D1b* present in 2174 for early flowering but Overland carried the photoperiod sensitive allele *PPD-D1a* present in Jagger for late flowering (Fig. 2d).

Plants homozygous for the Apogee *PPD-D1b* allele on average flowered 45.5 days after planting, whereas plants homozygous for the Overland *PPD-D1a* allele on average flowered at 51.8 days, and this difference of 6.3 days between the two alleles was significant (*p* < 0.01). The difference between the homozygous Apogee *PPD-D1b* allele and the heterozygous *PPD-D1a*/*PPD-D1b* alleles (48 days) was not significant (*p* = 0.201), but the difference between heterozygous plants and homozygous plants for the Overland *PPD-D1a* allele was significant (*p* < 0.05). These results indicated that *PPD-D1b* for insensitivity to photoperiod (hence early flowering) was dominant to *vrn-D3a* for photoperiod sensitivity (hence late flowering) as can be seen by the heterozygous alleles being not statistically different from the photoperiod insensitive homozygous plants when the population was tested under the long day conditions.

### Genetic effects of the four genes on flowering time

When the 858 F_2_ plants were genotyped, this population showed the expected 1:2:1 segregation ratio for each of the genotyped genes. Based on analysis of individual genes, the two homozygous classes of *vrn-A1*, *VRN-B1*, *vrn-D3*, and *PPD-D1* showed significant differences of 45.7, 15.4, 8.9 and 6.3 days respectively; therefore, the effect intensity of a single gene on the flowering time was ranked in the order of *vrn-A1 > VRN-B1 > vrn-D3 > PPD-D1*.

**Fig. 3:**
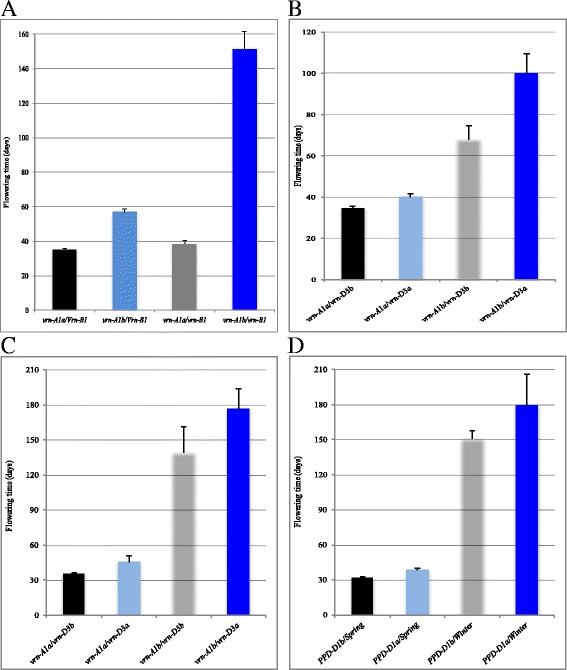
Interactive effects of flowering genes. **a** Interactions between *vrn-A1* and *VRN-B1*. Plants are classified into four genotypic classes and compared for flowering time, dominant *vrn-A1a* and dominant *Vrn-B1*, dominant *vrn-A1a* but recessive *vrn-B1*, recessive *vrn-A1b* but dominant *Vrn-B1*, and recessive *vrn-A1b* and recessive *vrn-B1*. **b** Interactions between *vrn-A1* and *vrn-D3*. All plants are classified into four genotypic classes regardless of allele status of *VRN-B1* or *PPD-D1*. **c** Interactions between *vrn-A1* and *vrn-D3* in the presence of recessive *vrn-B1* allele. All plants that have dominant *Vrn-B1* allele are classified into four genotypic classes and compared for flowering time, dominant *vrn-A1a* and dominant *vrn-D3a*, dominant *vrn-A1a* but recessive *vrn-D3b*, recessive *vrn-A1b* but dominant *vrn-D3a*, and recessive *vrn-A1b* and recessive *vrn-D3b*. **d** Effects of *PPD-D1* under different genetic backgrounds. Spring indicates the dominant allele for *vrn-A1a* and *Vrn-B1*, and winter indicates the recessive allele for *vrn-A1b* and *vrn-B1*

In addition to the accumulated effects of the four individual genes, the effects from the interactions between the four genes were analyzed in the F_2_ population using the factorial GLM ANOVA (Table 2). Two epistatic interactions were observed. The first epistatic interaction was between *vrn-A1* and *VRN-B1* (*p* = 6.83E-107). The epistatic interaction was analyzed using those plants that carried homozygous allele for *vrn-A1* and *VRN-B1*. In the presence of the dominant Apogee *Vrn-B1* allele, the flowering time of 56 plants carrying the Apogee *vrn-A1a* allele was 35 days after planting, whereas the flowering time of 43 plants carrying the Overland *vrn-A1b* allele and the dominant Apogee *Vrn-B1* genetic background was 56.8 days after planting, showing a difference of 21.8 days (Fig. 3a). In the presence of the recessive Apogee *vrn-B1* allele, the flowering time of 48 plants carrying the Apogee *vrn-A1a* allele was 38.6 days, whereas 27 plants carrying the Overland *vrn-A1b* allele had a flowering time of 151.6 days, showing a difference of up to 113 days. Similarly, the difference between the dominant Apogee *Vrn-B1* and the recessive Overland *vrn-B1* was 3.6 days in the presence of the dominant Apogee *vrn-A1a* allele, but it was 94.8 days between the two alleles in the presence of the recessive Overland *vrn-B1* allele (Fig. 3a).

**Table 2:** Analyses on interactions among *vrn-A1*, *VRN-B1*, *vrn-D3*, and *PPD-D1*

Source	DF	Sum of squares	Mean square	F Value	Pr > F	R2 (%)
*vrn-A1*	2	199,978.4759	99,989.2379	577.45	<.0001	38%
*VRN-B1*	2	40,238.9184	20,119.4592	116.19	<.0001	8%
vrn-A1*VRN-B1	4	128,602.4832	32,150.6208	185.67	<.0001	24%
*PPD-D1*	2	4085.9387	2042.9694	11.8	<.0001	1%
vrn-A1*PPD-D1	4	2213.353	553.3382	3.2	0.0129	0%
VRN-B1* PPD-D1	4	314.2033	78.5508	0.45	0.7698	0%
vrn-A1* VRN-B1* PPD-D1	8	3239.9174	404.9897	2.34	0.0174	1%
*vrn-D3*	2	4159.1734	2079.5867	12.01	<.0001	1%
vrn-A1*vrn-D3	4	4007.5511	1001.8878	5.79	0.0001	1%
VRN-B1* vrn-D3	4	1430.9048	357.7262	2.07	0.0835	0%
vrn-A1*VRN-B1* vrn-D3	8	3339.6495	417.4562	2.41	0.0142	1%
PPD-D1* vrn-D3	4	415.8344	103.9586	0.6	0.6625	0%
vrn-A1* PPD-D1* vrn-D3	8	1560.2604	195.0326	1.13	0.3429	0%
VRN-B1* PPD-D1* vrn-D3	8	944.0415	118.0052	0.68	0.7082	0%
vrn-A1* VRN-B1* PPD-D1* vrn-D3	14	2447.9668	174.8548	1.01	0.441	0%
Error	779	134,889.9808	173.1579			
Corrected Total	857	531,868.6527

The second epistatic interaction was between *vrn-A1* and *vrn-D3* (*p* = 6.33E-7). Due to the presence of interaction between *vrn-A1* and *VRN-B1*, the interactions *vrn-A1* and *vrn-D3* were analyzed under different *VRN-B1* genetic backgrounds. Regardless of the presence or absence of *VRN-B1*, the *vrn-D3* effect within the dominant Apogee *vrn-A1a* allele was 5.1 days (34.9 days for *vrn-D3a* in 56 plants compared with 40 days for *vrn-D3b* in 38 plants), which was significant (*p* < 0.01) (Fig. 3b). The *vrn-D3* effect within the recessive Overland *vrn-A1* genetic background was 31.8 days (68 days for *vrn-D3a* in 38 plants vs. 99.8 days for *vrn-D3b* in 34 plants), which was also significant (*p* < 0.01) (Fig. 3b). However, *vrn-A1* and *vrn-D3* interaction was not always detectable under different *VRN-B1* backgrounds. When the dominant *Vrn-B1* was present, the *vrn-D3* effect within the dominant Apogee *vrn-A1a* allele was 2.6 days (34.2 days for *vrn-D3a* vs. 36.8 days for *vrn-D3b*), which was not significant (*p* = 0.142). However, the *vrn-D3* effect within the recessive Overland *vrn-A1* genetic background was 14.1 days (51.1 days for *vrn-D3a* vs. 65.2 days for *vrn-D3b*), which was significant (*p* < 0.05). Conversely, when the dominant *Vrn-B1* was absent, the *vrn-D3* effect within the dominant Apogee *vrn-A1a* allele was 10.6 days (35 days for *vrn-D3a* vs. 45.6 days for *vrn-D3b*), which was significant (*p* < 0.05) (Fig. 3c). However, the *vrn-D3* effect within the recessive Overland *vrn-A1* genetic background was 37.3 days (139.4 days for *vrn-D3a* vs. 176.7 days for *vrn-D3b*), which was not significant (*p* = 0.206) due to a large variation in flowering time in winter plants without vernalization (Fig. 3c).

Significant interactions were also detectable among three genes, *vrn-A1, VRN-B1,* and *vrn-D3*, and among three genes, *vrn-A1, VRN-B1,* and *PPD-D1* (*p* = 0.0174). When both *vrn-A1a* and *Vrn-B1* were fixed at a dominant allele for spring type, the plants homozygous for the *PPD-D1b* allele flowered 31.8 days after planting and the plants homozygous for the *PPD-D1a* allele flowered 38.4 days after planting. The difference between the two alleles was 6.6 days and significant (*p* = 8.14E-05) (Fig. 3d). However, when both *vrn-A1b* and *vrn-B1* were fixed at a recessive allele for winter type, the plants homozygous for the *PPD-D1b* allele flowered 151 days after planting and *PPD-D1b* the plants carrying homozygous *PPD-D1a* allele flowered 179.2 days after planting. The difference between the two alleles was 28.2 days, but it was not significant (*p* = 0.392) again due to large variation among the plants behaving as winter wheat (Fig. 3d). Therefore, the effects of *PPD-D1* was dependent on the genetic background.

### Validation of the genetic effects of the genes/alleles on flowering time in F_3_ populations

**Fig. 4:**
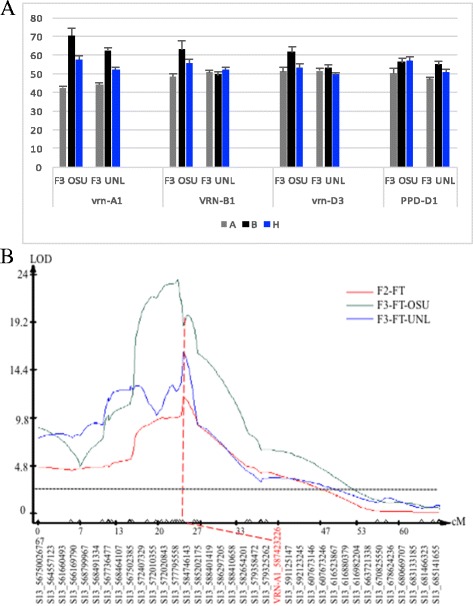
Mapping of flowering genes in Apogee x Overland populations. **a** Genetic effects of individual genes on flowering time in F_3_ populations. A indicates the Apogee allele, B indicates the Overland allele, and H indicates the heterozygous allele. **b** The major QTL at the *vrn-A1* locus. The number of a GBS marker indicates the physical location of the marker in the wheat genome sequence of Chinese Spring released in the International Wheat Genome Sequencing Consortium (IWGSC). The vertical dotted line indicates the logarithm of the odds (LOD) significance threshold of 2.5. The vrn-A1 gene assembled in this group is highlighted in red

A subset of 203 F_2:3_ lines were used to validate the genetic effects of the four flowering time. These F_3_ lines, each derived from the same F_2_ line, were tested in two different locations. The genetic effects of the *vrn-A1* gene on flowering time were observed in these F_3_ lines at both Oklahoma State University (OSU) and University of Nebraska in Lincoln (UNL) (Fig. 4a). However, the genetic effects of the *VRN-B1* gene and the *vrn-D3* gene were detectable at OSU but not at UNL, whereas the genetic effects of the *PPD-D1* gene were detectable at UNL but not at OSU (Fig. 4a).

The total effects of the four individual genes together with their interactive effects, explained approximately 75% of the total phenotypic variation in the F_2_ population (Table 2). To test if any other major genes affected the segregation of flowering time, 203 F_2:3_ lines were genotyped using genotyping-by-sequencing (GBS). A total of 61,217 SNP markers was identified in the population. Further, filtering with appropriate filtering criteria (see Methods) and polymorphism for both the parents, 1969 high-quality SNPs were used to develop linkage maps. These high-quality markers were then assembled into 22 linkage groups that were assigned to 21 chromosomes and an uncharacterized group. A major QTL peak at the *vrn-A1* gene was observed for flowering time in the F_2_ population and F_3_ populations (Fig. 4b). No other QTL was observed in the GBS-genotyped population.

## Discussion

The cumulative effects of the early alleles for the four flowering time genes result in the super short life cycle of Apogee.

Apogee wheat was found to have an early allele for all of four flowering genes identified in this study, as Apogee was crossed with winter cultivar Overland and the resulting F_2_ population was tested under constant warm temperature and long day conditions. The allele combination of the four genes resulted in a genotype that flowered as early as 25 days after planting. All four genes are related to the genes known for responses to vernalization and photoperiod, the two external cues that are regarded as the most important factors regulating wheat development.

However, it was surprising that that Apogee had the same *vrn-A1a* allele as observed in winter wheat cultivar Jagger. The flowering genes *VRN-A1* were cloned based on phenotypic differences between spring wheat carrying the dominant *Vrn-A1* and winter wheat carrying the recessive *vrn-A1* allele [10]. Moreover, *vrn-A1* was found to control variation in vernalization requirement duration between two winter wheat cultivars, Jagger carrying the *vrn-A1a* allele for less vernalization requirement and 2174 carrying the *vrn-A1b* for more vernalization requirement [23]. Surprisingly, Apogee was found to have the *vrn-A1a* allele for early flowering, which is the same allele as observed in Jagger, whereas Overland was found to have the *vrn-A1b* allele for late flowering, which is the same allele as observed in 2174. This observation also supported our report that the early flowering conferred by *vrn-A1* was due to the protein form of *vrn-A1a* but not due to two copies of *vrn-A1b* [23]. It would be intriguing to know how the vernalization requirement by the *vrn-A1a* allele in Apogee is overcome.

It was also surprising that Apogee had the same *vrn-D3a* allele as observed in winter wheat cultivar Jagger. The wheat *VRN-B3* gene was cloned using a population generated from two cultivars, CS and Hope, both of which have a dominant *Vrn-D1* allele, and the difference between the *Vrn-B3* and *vrn-B3* relies on an indel polymorphism in the promoter region involved in the regulation of the *VRN-B3* transcript levels [11]. Furthermore, *vrn-D3* was identified to be associated with allelic variation for flowering time among winter wheat cultivars [26–28]. The difference between the *vrn-D3a* in Apogee like Jagger and *vrn-D3b* in Overland like 2174 relies on a SNP that results in a frame shift involving 81 amino acids [26].

### Comparative effects of different flowering genes in the same population

This is the first study that four genes were analyzed for their effects on flowering time in the same population in hexaploid wheat. In the Apogee x Overland F_2_ population, a single *vrn-A1a* allele was able to flower as early as 30 days after planting. Due to the strongest effect of the *Vrn-A1a* allele, it has been incorporated into spring wheat cultivars in Canadian breeding programs to provide frost avoidance in short-season environments [31]. Conversely, a deletion of *TmVRN1* in *T. monococcum* results in a never-flowering wheat plant (mvp) [32]. These studies have demonstrated the powerful effect of *VRN-A1* on flowering time in both spring wheat and winter wheat backgrounds.

The comparative effects of different genes on flowering time in wheat were reported in previous studies. The effect intensity of the three *VRN-1* genes was ranked as *Vrn-A1* > *Vrn-B1* > *Vrn-D1*, based on analyses on Triple Dirk isogenic lines [33] and in Chinese wheat cultivars [34]. This study presented experimental evidence for the greater effect of *vrn-A1a* than *Vrn-B1* on flowering time in a single segregating population.

### Allelic variation in *vrn-A1a* and its epistatic interaction with *Vrn-B1*

In a previous study, the spring and dominant allele *Vrn-A1a* was reported to cause earlier transcription of the recessive *vrn-B1* allele in the Triple Dirk line TDD, forming of a positive feedback loop that coordinates the transcription of the different *VRN-1* alleles during flowering initiation [35]. In this study, the winter but dominant *vrn-A1a* allele was also epistatic to *VRN-B1*. The effect of *VRN-B1* on flowering time was 3.6 days in the presence of the Apogee *vrn-A1a* allele (*vrn-A1a/Vrn-B1* vs. *vrn-A1a/vrn-B1*) but this effect was increased to 94.8 days in the presence of the Overland *vrn-A1b* allele (*vrn-A1b/Vrn-B1* vs. *vrn-A1b/vrn-B1*). The effect of the dominant *Vrn-B1* allele on *vrn-A1* was also observed in the phenotype. The effect of *vrn-A1* on flowering time was 21.8 days in the presence of the Apogee *Vrn-B1* allele (*vrn-A1a/Vrn-B1* vs. *vrn-A1b/Vrn-B1*) but this effect was increased to 113 days in the presence of the Overland *vrn-B1* allele (*vrn-A1a/vrn-B1* vs. *vrn-A1b/vrn-B1b*). Since the plants carrying the dominant *vrn-A1a* allele but the recessive *vrn-B1* allele had earlier flowering time (35 days) than the plants carrying the dominant *Vrn-B1* allele but the recessive *vrn-A1b* allele, it is more likely that *vrn1-A1a* was epistatic to *VRN-B1* in the Apogee and Overland F_2_ population. This study indicated that the accelerated effect of the *vrn-A1a* allele on flowering time was detectable under different genetic backgrounds in wheat.

### Accelerated effect of the dominant *PPD-D1b* and *vrn-3a* allele for flowering but not for the developmental transition

Vernalization and photoperiod genes are believed to interactively regulate flowering time in temperate cereal crops [27, 36–40], but no direct evidence is available for interactions of multiple flowering time genes in hexaploid wheat. Results in this study indicated that *PPD-D1b*, which is insensitive to photoperiod, can be used to accelerate flowering in spring plants that have been induced to flower by *vrn-A1a* or *Vrn-B1*. However, no significant effect was observed when *PPD-D1b* was placed in the genetic backgrounds of recessive *vrn-A1b* and *vrn-B1*. When *vrn-A1a* and *Vrn-B1* were fixed in a plant for an early flowering allele, the promotion effect of the *vrn-D3a* allele was not detectable, because this plant flowered extremely early already. When *vrn-A1b* and *vrn-B1* were fixed in a plant for a late flowering allele, the effect of the *vrn-D3a* allele was not significant either, because *vrn-D3a* alone was insufficient to initiate the transition from the vegetative to reproductive development in winter wheat without vernalization. The *vrn-D3* haplotype was functionally characterized in the Jagger × 2174 RIL population, in which that *vrn-D3*a was found to have accelerated flowering and physiological maturity dates but no significant effect on initial stem elongation, a trait best representing the developmental transition from vegetative to reproductive development [40]. This conclusion was also true in another winter wheat population generated from two winter cultivars Intrada and Cimarron [27]. The same *vrn-D3* haplotype was also reported to have significant effect on flowering time in a large collection of diverse germplasm [26].

**Fig. 5:**
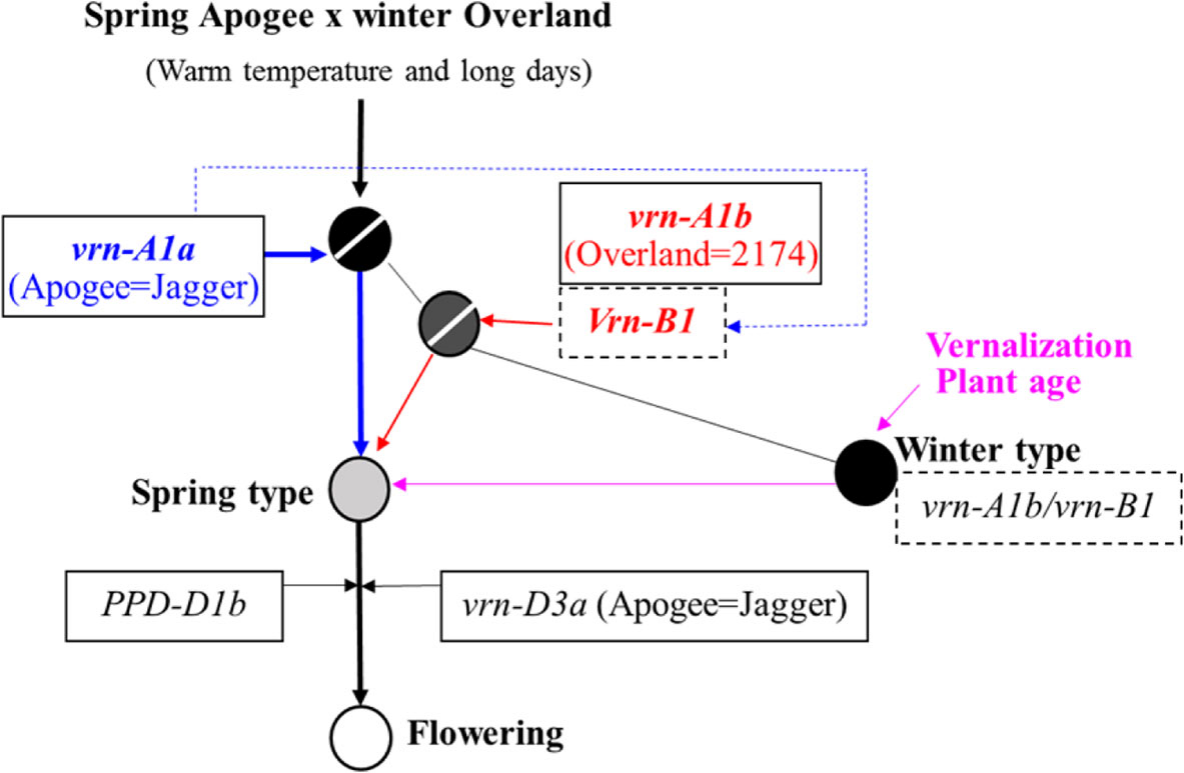
An updated genetic model for the wheat flowering pathway. Thin arrows indicate promotion. When grown under warm temperature and long day condition, the Apogee x Overland F_2_ population are segregated to into different genotypes; the plants that carry dominant *vrn-A1a* allele will behave as spring type and flower first (flowering signals transited in the pathway indicted in blue), the plants that carry dominant *Vrn-B1* allele will behave as spring type and flower later (flowering signals transited in the pathway indicated in red), because *Vrn-B1* has less power than *vrn-A1a*, and *Vrn-B1* is promoted by *vrn-A1a* in a positive feedback loop; the plants that carry recessive alleles for both *vrn-A1b* and *vrn-B1* will behave as winter type and will not flower unless vernalization is provided or flower with plant age. *PPD-D1b* and *vrn-D3a* will accelerate flowering in the spring plants that have been induced to flower by *vrn-A1a*/*Vrn-B1* or the winter plants that have been vernalized

Based on these observations, a model for regulating node sites of the four flowering genes in the vernalization pathway is established (Fig. 5). In this model, *vrn-A1a* replaces vernalization to directly induce the developmental transition. *Vrn-B1* is able to induce this transition, but it acts later than *vrn-A1a* and is also accelerated by *vrn-A1a* due to the presence of a positive feedback loop. Under the experimental conditions tested in this study, *PPD-D1b* and *vrn-D3a* are able to accelerate flowering only after the developmental transition has been initiated by *vrn-A1a* or *Vrn-B1* in spring plants. In this model, *vrn-D3a* acts in the downstream of *Vrn-A1*, which seems contradictory to a previous model, in which *Vrn-A1* acts in the downstream of *Vrn-B3* in the vernalization pathway [11]. However, *vrn-D3a* and *Vrn-B3* may have different mechanisms in regulation of flowering time under different genetic backgrounds in wheat.

## Conclusion

Apogee wheat is able to flower one month after planting and without vernalization requirement, so each generation will be 2 months or less and we can grow up to 6 generations per year which will greatly shorten the breeding cycle. Conversely, a winter wheat cultivar takes at least 4 to 5 months per generation, so only two (or at most three) generations can be grown per year. When winter wheat cultivars are crossed and backcrossed with Apogee, the resulting backcrossed lines will be selected for reduced generation time using molecular tools for the Apogee alleles for the four flowering genes. Such rapid cycling lines will not require vernalization to induce flowering and will be selected for traits of interest such as disease or pest resistance, high yield, or end-use quality. The elite breeding lines will then be crossed to the winter wheat recurrent parent used to create the rapid cycling lines to recover the winter growth habit, thus creating adapted backcross derived lines quickly and efficiently, and accelerating winter wheat breeding schemes.

## Methods

Apogee (PI 592742) is a full-dwarf hard red spring wheat cultivar (pedigree: Parula/Super dwarf) and released by the Utah Agric. Exp. Station in cooperation with NASA in 1996. ‘Overland’ (PI 647959) is hard red winter wheat [pedigree: Millennium sib//(ND8974) Seward/Archer)] that was developed by the Nebraska Agriculture Experiment Station and released in 2007. Apogee and Overland were tested three times in a greenhouse conditioned with long days (16 h for light at 25 ± 2 °C, and 8 h for darkness at 20 ± 2 °C) during the whole life cycle, located on the Stillwater campus of Oklahoma State University (OSU). In addition to natural sunlight, high-pressure sodium lamps were used to provide supplemental lighting for nights and cloudy days. Apogee flowered about 30 days after planting, whereas Overland did not flower within 6 months unless it was vernalized. Overland was vernalized at 4 °C and under long days. Apogee was crossed with Overland to create 20 hybrid seeds. The F_1_ seeds were self-pollinated and used to generate a population of 858 F_2_ plants.

All of the F_2_ plants were not vernalized to identify genes associated with early flowering genes in Apogee without requirement of vernalization. An F_2_ population consisting of up to 858 plantswas tested in this study, and the large population size should have replication of each genotypic class, necessary for statistical comparisons. A total of 203 F_2:3_ lines were planted in February 2015 and tested at OSU and University of Nebraska in Lincoln (UNL) to validate the effects of flowering time genes. The flowering time of a plant was recorded when the first spike of the plant was emerging from the flag leaf sheath of the main stem or a primary tiller. Flowering time was scored for each plant of the population.

Genomic DNA was extracted from parental lines and the 858 F_2_ individual plants using the method as previously described [41]. PCR markers for genes known to regulate flowering time were used to identify polymorphism between Apogee and Overland, and primer sets used for the gene markers are listed in Additional file 1: Table S1. The protocols for PCR markers for each gene were cited from previous studies. Those markers that showed allelic variation between Apogee and Overland were used to genotype 858 F_2_ plants, and those markers that showed no difference were not further analyzed. Details for the four genes that showed allelic variation between Apogee and Overland are elaborated below for convenience.

### VRN-A1

Five pairs of primers were used to test polymorphisms in *VRN-A1* between Apogee and Overland. The first primer set, VRN1AF/VRN1R, was used to detect indel polymorphisms in the *VRN-A1* promoter region. The second and third primer sets were used to detect the indel polymorphism in intron 1 in *VRN-A1*, Ex1/C/F//Intr1/A/R3 for the deletion in the *Vrn-A1c* allele and Intr1/C/F//Intr1/AB/R for the non-deletion in the *vrn-A1* allele [24]. The fourth and fifth primer sets were used to detect the difference in coding region, VRN-A1F4/VRN-A1R42 for a SNP in exon 4 [40] and VRN-A1F7B/VRN-A1R7 for a SNP in exon 7 [23].

### VRN-B1

Two pairs of primers were used to detect the indel polymorphism in intron one in *VRN-B1*, Intr1/B/F//Intr1/B/R3 for the deletion in the *Vrn-B1* allele and Intr1/B/F//Intr1/B/R4 for the non-deletion in the *vrn-B1* allele [24]. Primers Intr1/B/F//Intr1/B/R3 amplified products with a band of 709 bp, and Intr1/B/F//Intr1/B/R4 amplified products with a band of 1149 bp. The forward primer and the two reverse primers for the same progeny sample were used to amplify the homozygous or heterozygous *VRN-B1* alleles in two different reactions, but the PCR products were electrophoresed in the same gel well.

### VRN-D3

The primer set VRN-D3-F6/VRN-D3-R8 was used to test allelic variation in *VRN-D3*. Products were digested with restriction enzyme *Nco*I, producing polymorphic DNA fragments due to a 1 bp indel or (G)_3or4_ difference in *VRN-D3* coding region, 375 bp and 27 bp fragments for *vrn-D3a* allele and 401 bp for the *vrn-D3b* allele [28].

### PPD-D1

One forward primer PPD-D1_F was paired with two reverse primers PPD-D1_R1 and PPD-D1_R2 to simultaneously amplify two PPD alleles in a one shot PCR reaction [14]. One allele is for deletion, producing a 288 bp fragment from the photoperiod insensitive allele, and the other is for non-deletion, producing a 414 bp fragment from the photoperiod sensitive allele.

Real time-PCR was used to determine *VRN-A1* copy number by the SYBR Green PCR Master Mix, and *TaCO2* was used as an endogenous control. Primers for *vrn-A1* and for *TaCO2* control were cited from previous studies [35].

The gene interactions were assessed using the Factorial analysis of variance (ANOVA) of the General Linear Model (GLM) in SAS 9.4 (SAS Institute, Raleigh, NC). The epistatic interactions between two loci were analyzed using the program EPISTACY in SAS 9.4 [42].

The 203 F_3_ lines were genotyped using GBS at the Wheat Genetics and Germplasm Improvement Laboratory at Kansas State University (http://www.wheatgenetics.org). The SNP calls were made using the TASSEL v5.2.30 software (Glaubitz et al. 2014) with default setting except for two parameters to increase the SNP calling stringency. First, minimum quality score within the barcode to be accepted was changed from default value of 0 to 20. Second, minimum count of reads for a tag to be output for alignment against the reference genome was changed from default value of 1 to 5. The reference genome v0.4 of the bread wheat variety ‘Chinese spring’ was utilized for making SNP calls. The SNP markers were filtered to exclude markers with missing percentage greater than 20% and minor allele frequency less than 0.2. The SNPs were then converted from a nucleotide-based format to parent-based format using GenosToABHPlugin in TASSEL for linkage map development. Based on the conserved locations of the GBS SNP markers, these linkage groups were assigned to 19 of the 21 chromosomes in hexaploid wheat. On the basis of whole-genome QTL scanning using Interval Mapping (IM) analysis, the QTL for flowering time was screened.

Terms for genes/alleles are referred to, *VRN-A1* for a common allele for either dominant or recessive allele, *Vrn-A1* for the dominant allele in spring wheat, *vrn-A1a* for a recessive allele present in winter wheat Jagger for early flowering and *vrn-A1b* for a recessive allele present in winter wheat 2174 for late flowering; *Vrn-3* for the dominant allele in spring wheat, *vrn-D3a* for a recessive allele present in Jagger for early flowering and *vrn-A1b* for a recessive allele present in 2174 for late flowering; *Vrn-B1* for early flowering in spring wheat, and *vrn-B1* for late flowering in winter wheat; *PPD-D1a* for photoperiod sensitive and late flowering in Jagger and *PPD-D1b* for photoperiod insensitive and early flowering in 2174.

## Additional file

Additional file 1: Table S1. Primers used in PCR reactions for identification of allelic variation at vrn-A1, VRN-B1, vrn-D3, and PPD-D1. (DOCX 18 kb)

## Abbreviations

EPS: Earliness per se (EPS)
GBS: Genotyping-by-sequencing
MITE: Miniature inverted-repeat transposable element
PCR: Polymerase chain reaction
PPD: Photoperiod response
SNP: Single nucleotide polymorphism
VRN: Vernalization
