# Supplementary material for: Genetic basis of the very short life cycle of ‘Apogee’ wheat
Source: BMC Genomics. 2017 Oct 31;18:838. doi: 10.1186/s12864-017-4239-8 (PMC5664786; doi:10.1186/s12864-017-4239-8)
Supplement: Additional file 1: Table S1. — Primers used in PCR reactions for identification of allelic variation at vrn-A1, VRN-B1, vrn-D3, and PPD-D1. (DOCX 18 kb) [file 12864_2017_4239_MOESM1_ESM.docx]

**Table S1. Primers used in PCR reactions for identification of allelic variation at *vrn-A1*, *VRN-B1*, *vrn-D3*, and *PPD-D1***

| Allele | Mutation site | Primers | sequence (5'-3') | Expected size (bp) | Tm ^a^ | Reference |
| --- | --- | --- | --- | --- | --- | --- |
| *Vrn-A1* | Indel in promoter | VRN1AF | GAAAGGAAAAATTCTGCTCG | 484  /Insertion | 50 | Yan, et al., 2004 |
|  |  | VRN1R | TGCACCTTCCCCCGCCCCAT |  |  |  |
| *Vrn-A1c* | Deletion in intron 1 | Ex1/C/F | GTTCTCCACCGAGTCATGGT | 522 | 55.6 | Fu et al., 2005 |
|  |  | Intr1/A/R3 | AAGTAAGACAACACGAATGTGAGA |  |  |  |
| *vrn-A1* | Non-deletion in intron 1 | Intr1/C/F | GCACTCCTAACCCACTAACC | 1068 | 56 | Fu et al., 2005 |
|  |  | Intr1/AB/R | TCATCCATCATCAAGGCAAA |  |  |  |
| *vrn-A1a* | SNP in exon 7 | VRN-A1F7B | GTGGAGAAGCAGAAGGCGCATG | 221 | 55 | Li et al., 2013 |
|  |  | VRN-A1R7 | CCGACAGAACTGCATAGAGACC |  |  |  |
| *vrn-A1a* | SNP in exon 4 | VRN-A1-F4 | CAACTTGTTTGGGACTAAAGGC | 375 | 55 | Chen et al., 2009 |
|  |  | VRN-A1-R42 | CTGCAACTCCTTGAGATTCAAAG |  |  |  |
| *Vrn-B1* | Deletion in intron 1 | Intr1/B/F | CAAGTGGAACGGTTAGGACA | 709 | 58 | Fu et al., 2005 |
|  |  | Intr1/B/R3 | CTCATGCCAAAAATTGAAGATGA |  |  |  |
| *vrn-B1* | Non-deletion in intron 1 | Intr1/B/F | CAAGTGGAACGGTTAGGACA | 1149 | 56.4 | Fu et al., 2005 |
|  |  | Intr1/B/R4 | CAAATGAAAAGGAATGAGAGCA |  |  |  |
| *Vrn-D1* | Deletion in intorn 1 | Intr1/D/F | GTTGTCTGCCTCATCAAATCC | 1671 | 61 | Fu et al., 2005 |
|  |  | Intr1/D/R3 | GGTCACTGGTGGTCTGTGC |  |  |  |
| *vrn-D1* | Non-deletion in intron 1 | Intr1/D/F | GTTGTCTGCCTCATCAAATCC | 997 | 61 | Fu et al., 2005 |
|  |  | Intr1/D/R4 | AAATGAAAAGGAACGAGAGCG |  |  |  |
| *vrn-D3a* | 4G in exon 3 | VRN-D3-F6 | CTTCTATTCACATGTTTCGTTCATG | 401 | 55 | Chen et al., 2010 |
|  |  | VRN-D3-R8 | ACGAGCACGAAGCGATGGATCGC |  |  |  |
| *HOX-B1* | SNP in exon | HOX-6BF1 | GCGGCGCGCCAAGCTGGAC | 317 | 55 | Li et al., 2013 |
|  |  | HOX-6BC1R2M | CAGCTGCACATCGAGCAGACAC |  |  |  |
| *PPD-D1* | Indel in promoter | PPD-D1_F | ACGCCTCCCACTACACTG | 288/414 | 54 | Beales et al., 2007 |
|  |  | PPD-D1_R1 | GTTGGTTCAAACAGAGAGC |  |  |  |
|  |  | PPD-D1_R2 | CACTGGTGGTAGCTGAGATT |  |  |  |

^a^ annealing temperature in PCRs.
